# Supplementary material for: Do chimpanzees (Pan troglodytes) attribute preferences to virtual competitors?
Source: PLoS One. 2025 Sep 9;20(9):e0329468. doi: 10.1371/journal.pone.0329468 (PMC12419670; doi:10.1371/journal.pone.0329468)
Supplement: S1 Table — (DOCX) [file pone.0329468.s001.docx]

| Subject | Test | | Control | |
| --- | --- | --- | --- | --- |
|  | % trials correct | binomial result | % trials correct | binomial result |
| Azibo | 49.0 | p=.920 | 45.0 | p=.368 |
| Fraukje | 51.0 | p=.920 | 49.0 | p=.920 |
| Riet | 45.0 | p=.368 | 46.0 | p=.484 |
| Swela | 49.0 | p=.920 | 51.0 | p=.920 |
| Tai | 54.0 | p=.484 | 54.0 | p=.484 |
| Youma | 43.3 | p=.246 | 44.8 | p=.391 |

**S1 Table. Experiment 1 Observed Success Rates and Binomial Tests.**
