# Supplementary material for: Do chimpanzees (Pan troglodytes) attribute preferences to virtual competitors?
Source: PLoS One. 2025 Sep 9;20(9):e0329468. doi: 10.1371/journal.pone.0329468 (PMC12419670; doi:10.1371/journal.pone.0329468)
Supplement: S2 Table — (DOCX) [file pone.0329468.s002.docx]

| Subject | % trials went Left | % trials went Right | Side Bias |
| --- | --- | --- | --- |
| Azibo | 16.0 | 84.0 | R, p<.01 |
| Fraukje | 29.0 | 71.0 | R, p<.01 |
| Riet | 87.5 | 12.5 | L, p<.01 |
| Swela | 80.0 | 20.0 | L, p<.01 |
| Tai | 62.0 | 38.0 | L, p<.01 |
| Youma | 35.6 | 64.4 | R, p<.01 |

**S2 Table. Experiment 1 Individual Subject Side Biases (Binomial Tests).**
