# Supplementary material for: Do chimpanzees (Pan troglodytes) attribute preferences to virtual competitors?
Source: PLoS One. 2025 Sep 9;20(9):e0329468. doi: 10.1371/journal.pone.0329468 (PMC12419670; doi:10.1371/journal.pone.0329468)
Supplement: S3 Table — (DOCX) [file pone.0329468.s003.docx]

| Subject | Test | | Control | |
| --- | --- | --- | --- | --- |
|  | % trials chose rabbit/% trials chose dog | Prey Bias | % trials chose chicken/% trials chose antelope | Prey Bias |
| Azibo | 53.0/47.0 | None, p=.61 | 58.0/42.0 | None, p=.133 |
| Fraukje | 53.0/47.0 | None, p=.61 | 79.0/21.0 | Chicken, p<.01 |
| Riet | 47.0/53.0 | None, p=.61 | 50.0/50.0 | None, p=1 |
| Swela | 51.0/49.0 | None, p=.92 | 55.0/45.0 | None, p=.368 |
| Tai | 54.0/46.0 | None, p=.48 | 50.0/50.0 | None, p=1 |
| Youma | 68.9/31.1 | Rabbit, p<.01 | 67.8/32.2 | Chicken, p<.01 |

**S3 Table. Experiment 1 Individual Subject Prey Biases (Binomial Tests).**
