# Supplementary material for: Do chimpanzees (Pan troglodytes) attribute preferences to virtual competitors?
Source: PLoS One. 2025 Sep 9;20(9):e0329468. doi: 10.1371/journal.pone.0329468 (PMC12419670; doi:10.1371/journal.pone.0329468)
Supplement: S4 Table — (DOCX) [file pone.0329468.s004.docx]

| Subject | Test | | Control | |
| --- | --- | --- | --- | --- |
|  | % trials correct | binomial result | % trials correct | binomial result |
| Alex | 51 | p=.920 | 57 | p=.193 |
| Carola | 40 | p=.057 | 50 | p=1 |
| Changa | 43 | p=.193 | 46 | p=.484 |
| Corrie | 55 | p=.368 | 50 | p=1 |
| Daza | 48 | p=.764 | 48 | p=.764 |
| Frederike | 47 | p=.617 | 47 | p=.617 |
| Hope | 51 | p=.920 | 51 | p=.920 |
| Sandra | 36 | p<.01 | 49 | p=.920 |
| Zira | 49 | p=.920 | 44 | p=.271 |

**S4 Table. Experiment 2 Observed Success Rates and Binomial Tests.**
