# Supplementary material for: Do chimpanzees (Pan troglodytes) attribute preferences to virtual competitors?
Source: PLoS One. 2025 Sep 9;20(9):e0329468. doi: 10.1371/journal.pone.0329468 (PMC12419670; doi:10.1371/journal.pone.0329468)
Supplement: S5 Table — (DOCX) [file pone.0329468.s005.docx]

| Subject | % trials went Right | % trials went Left | Side bias |
| --- | --- | --- | --- |
| Alex | 57.0 | 43.0 | None, p=.056 |
| Carola | 23.0 | 77.0 | L, p<.01 |
| Changa | 25.0 | 75.0 | L, p<.01 |
| Corrie | 44.5 | 55.5 | None, p=.137 |
| Daza | 33.0 | 67.0 | L, p<.01 |
| Frederike | 49.5 | 50.5 | None, p=.944 |
| Hope | 50.0 | 50.0 | None, p=1 |
| Sandra | 29.0 | 71.0 | L, p=<.01 |
| Zira | 50.0 | 50.0 | None, p=1 |

**S5 Table. Experiment 2 Individual Subject Side Biases (Binomial Tests).**
