# Supplementary material for: Do chimpanzees (Pan troglodytes) attribute preferences to virtual competitors?
Source: PLoS One. 2025 Sep 9;20(9):e0329468. doi: 10.1371/journal.pone.0329468 (PMC12419670; doi:10.1371/journal.pone.0329468)
Supplement: S6 Table — (DOCX) [file pone.0329468.s006.docx]

| Subject | Test | | Control | | |
| --- | --- | --- | --- | --- | --- |
|  | % trials chose rabbit/% trials chose boar | Prey bias | % trials chose chicken/% trials chose antelope | Prey bias |  |
| Alex | 65/35 | Rabbit, p<.01 | 77/23 | Chicken, p<.01 |  |
| Carola | 74/26 | Rabbit, p<.01 | 40/60 | None, p=.057 |  |
| Changa | 45/55 | None, p=.368 | 68/32 | Chicken, p<.01 |  |
| Corrie | 71/29 | Rabbit, p<.01 | 45/55 | None, p=.368 |  |
| Daza | 29/71 | Boar, p<.01 | 31/69 | Antelope, p<.01 |  |
| Frederike | 89/11 | Rabbit, p<.01 | 83/17 | Chicken, p<.01 |  |
| Hope | 5/95 | Boar, p<.01 | 10/90 | Antelope, p<.01 |  |
| Sandra | 56/44 | None, p<.01 | 80/20 | Chicken, p<.01 |  |
| Zira | 14/86 | Boar, p<.01 | 28/72 | Antelope, p<.01 |  |

**S6 Table. Experiment 2 Individual Subject Prey Biases (Binomial Tests).**
