# Supplementary material for: Do chimpanzees (Pan troglodytes) attribute preferences to virtual competitors?
Source: PLoS One. 2025 Sep 9;20(9):e0329468. doi: 10.1371/journal.pone.0329468 (PMC12419670; doi:10.1371/journal.pone.0329468)
Supplement: Attachment — Submitted filename: revision_PONE-Chimpanzee.docx [file pone.0329468.s009.docx]

This study investigated the ability of chimpanzees to attribute preferences to virtual competitors and adjust their behavior accordingly. Two experiments were conducted, but no statistically significant results were found regarding the use of memory about competitors' preferences. Some behavioral aspects inherent to social animals raised concerns, as there could be associative learning where chimpanzees adjust their responses based on previous trials. To control for this, the study included control conditions where no information about competitors' preferences was provided. The results showed no significant differences between the test and control conditions, suggesting that chimpanzees were not using preference information to guide their choices.

**Major Comments:**

The introduction is excessively long and becomes tedious due to unnecessary detail. It could be improved by summarizing all examples or focusing only on the discrepancies—below, I provide some examples of how to condense it. While I understand the purpose of the study, I suggest that, given the extensive context and discrepancies regarding these behavioral aspects, the authors should clearly define a couple of specific objectives and formulate explicit hypotheses.

I find the procedure difficult to follow. Perhaps the authors could add a graphical representation of the procedure, explaining the stages and specifying the number of trials in each experiment so that the reader can properly follow the procedure and understand the number of trials, experimental units, and test runs.

My main concern is that the structure of the article does not align with *PLOS ONE*’s formatting. The authors present the Methods, Results, and Discussion sections separately for each experiment. I disagree with this approach because it becomes repetitive. The authors should restructure the manuscript so that I can better focus on the study's scientific contributions.

**Specific Comments:**

- **Lines 60-80**: Specify which of these studies involved experiments with virtual animals or clarify the conditions in which they were conducted.
- **Lines 44-60**: This paragraph provides context about multilevel societies in chimpanzees and the importance of avoiding competition. It could be condensed by removing specific examples and focusing on the main idea.
- **Lines 61-66**: This paragraph provides an example of a previous study on chimpanzees. Consider summarizing it, keeping only the key idea and removing unnecessary study details.
- **Lines 67-72**: This paragraph describes chimpanzees' behavior in competitive situations. It could be synthesized to focus on the relevance of understanding competitors' preferences without including excessive examples.
- **Lines 81-102**: This section provides background on competition in chimpanzees and the importance of understanding how they interpret others' preferences. It could be condensed to highlight the relevance of the research without excessive details on previous studies.
- **Lines 103-148**: This section analyzes prior research on primate competitive behavior. Summarizing key findings while mentioning only the most relevant results would help clarify the purpose of this study.
- **Lines 161-191**: This section justifies the chosen experimental approach. The justifications could be condensed, focusing on the main rationale behind the design choices and eliminating redundancies.
- **Lines 192-204**: The authors provide excessive details that belong in the Methods section—remove them. This section should include a couple of specific objectives (one for each experiment) and the hypotheses to be tested, given the extensive background provided.

**Methods:**

- **Line 207**: The *Methods* section should be placed first.
- **Line 228**: Specify the number of trials per individual.
- Many paragraphs are too short and disconnected, e.g., lines **278-281** and **308-310**—merge them with the following paragraphs.
- **Table 1**: I do not understand this table. A diagram of the procedure with images and connecting lines illustrating the experiment’s structure would be more effective.
- **Line 432**: Software should be mentioned at the end of the section, along with references for any statistical packages used.
- **Line 439**: The authors introduce hypotheses here—why were they not stated in the introduction?
- **Line 444**: This paragraph stands alone without context. Specify the statistical packages used and cite them. Also, indicate whether the data met normality assumptions and explain why a paired *t*-test was used—while it is known that paired tests are for related samples, the reader needs clarification. Explain what test was used when the data were non-normal. Additionally, in the binomial test, specify what constitutes success and what constitutes failure, as this distinction affects the formula's properties and the hypothesis test. Further justification for the 50% assumption in the formula is needed—this seems arbitrary, and references should support it. Otherwise, a *χ²* goodness-of-fit test might be more appropriate for frequency data.
