# Supplementary material for: Do chimpanzees (Pan troglodytes) attribute preferences to virtual competitors?
Source: PLoS One. 2025 Sep 9;20(9):e0329468. doi: 10.1371/journal.pone.0329468 (PMC12419670; doi:10.1371/journal.pone.0329468)
Supplement: Attachment — Submitted filename: Revisions.docx [file pone.0329468.s011.docx]

This study investigated the ability of chimpanzees to attribute preferences to virtual competitors and adjust their behavior accordingly. Two experiments were conducted, but no statistically significant results were found regarding the use of memory about competitors' preferences. The study provides valuable insights into the limitations and challenges in interpreting their social cognition. Additionally, the authors demonstrated significant effort in improving the experimental design, carefully attending to all our suggestions with great dedication. Overall, this work represents an important contribution to understanding chimpanzee cognitive abilities, showcasing methodological rigor and an openness to future research in this field. I have a minor comments, considering them, an additional revision is not required.

Minor

Line 86–89: Merge this paragraph with the previous one.
Table 1: Explain what “VR” means in the table title.
Line 407: *P* should be uppercase and italicized; include the degrees of freedom.
Line 452: I understand that “real world” refers to real-life conditions as opposed to virtual ones, but it would be helpful for readers if this were specified in parentheses.
Line 573: It’s good that it doesn’t sound repetitive—this was my suggestion—but I can’t imagine reading such a simplistic sentence in a scientific article. I suggest explicitly stating which part of the experiment is the same.
Line 648: Move the sentence “All analysis was conducted in R version 4.1.2 (25).” to the end of the paragraph.
Line 665: Does “(8)” refer to the degrees of freedom? *P* should be uppercase and italicized. Please check the rest as well.
Line 800: Explicitly state whether the study supported the hypothesis or not.
The figures are of very poor quality, though this is likely due to the proofing process.
The videos are wonderful.
